# Field study on the improvement of coal gangue filling using dynamic compaction

**DOI:** 10.1371/journal.pone.0250961

**Published:** 2021-05-05

**Authors:** Qingfeng Zhang, Dongquan Wang

**Affiliations:** 1 School of Mechanics and Civil Engineering, China University of Mining and Technology, Xuzhou, Jiangsu, China; 2 School of Environmental and Civil Engineering, Jiangnan University, Wuxi, Jiangsu, China; University of Genova, ITALY

## Abstract

In this study, dynamic compaction method was used to treat the gangue hill of the Xinglongzhuang coal mine in China, and the deep compaction of deep coal gangue was examined. The crushing characteristics and improving depth of coal gangue filling under different dynamic compaction conditions were determined. Dynamic compaction tests with different tamping energy were performed to improve the coal gangue filling. In addition, dynamic penetration tests and the foundation bearing capacity were conducted. The relationship between the tamping energy and improvement was investigated, and the optimum tamping energy, number of tamping blows, improving depth, and other dynamic compaction parameters and filling bearing characteristics were obtained. The field test results show that with increasing number of tamping blows, compaction induced deformation gradually decreased and begins to stabilize, while the optimum number of tamping blows increases with increasing ramming energy. The optimum number of tamping blows is in the range 9–11, and the effective coal gangue improving depth is in the range 6–8m, when the tamping energy is greater than 3000 kN.m. The gradation improved, and the weight percentage of the particles smaller than 4.75 mm was larger than 50%, resulting in better physical and mechanical behavior of the coal gangue filling. The coal gangue filling bearing capacity and anti-deformation ability increase with increasing tamping energy. The coal gangue filling bearing capacity reached at least 350 kPa after being improved by dynamic compaction with a tamping energy greater than 3000kN.m.

## 1. Introduction

Coal gangue is a solid waste generated during coal mining. It not only occupies large areas of land, but also causes significant amounts of pollution, which can seriously affect human health [1–3]. Therefore, proper disposing of coal gangue has become a major concern. Research indicates that coal gangue can be used in civil building materials such as landfill liner materials, pavement material admixture, and brick-making, etc [4–7].

Dynamic compaction (DC) method is a type of foundation treatment method pioneered by French engineer Menard in 1969. With improving construction methods and drainage conditions, DC method has been applied to reinforce sand and gravel soil, collapsible loess, miscellaneous filling, and other soils. With advantages of simple equipment, convenient construction, economical operation, remarkable effect, and saving material, the widely used DC method is relatively simple, convenient, and economical compared to other methods [8]; however, the design of DC method remains in the empirical stage [9–12]. Significant work remains to be done to improve the design of DC method.

In recent years, some scholars carried out field tests on different soils used to reinforce foundations using the DC method. Rollins, Feng *et al*., Wang *et al*., and Yang *et al*. [13–16] studied the reinforcement of collapsible loess using dynamic compaction. Field tests were conducted to determine the optimum DC operational parameters. The standard and static penetration tests and plate load tests were performed to evaluate the final effect of DC. The collapsibility was also measured before and after DC. Wu *et al*., Luo *et al*., Mei *et al*., and Gao *et al*. [17–20] studied the reinforcement of broken stone fills using DC. A suggestion for the best layer thickness for DC of fill was proposed. Shen *et al*. and Wang *et al*. [21, 22] studied the reinforcement of liquefied soil sing dynamic compaction. The research results will provide some guidance for the consolidation construction of liquefied soil foundations. Zhou *et al*. [23] improved saturated foundation based on the fluid–solid coupled method with soil cap model. Wang *et al*. [24] carried out an experimental study on the effect of fine content on dynamic compaction grouting in a completely decomposed granite in Hong Kong. Wang *et al*. [25] suggested an estimation method for ground deformation of granular soils caused by DC. Feng *et al*. [26–31] conducted field studies on dynamic consolidation of different types of soils.

Many domestic mining areas have begun to fill the subsidence area with coal gangue and then use it as a building foundation. This is inseparable from the series of advantages of coal gangue foundation. First of all, the use of coal gangue foundation can alleviate the shortage of construction land in mining areas and promote the sustainable development of the coal industry. Secondly, the use of coal gangue foundation can save land and protect the ecological environment. Finally, the use of coal gangue foundation has the advantages of low cost and saving project cost. The land resources in China are tight, and land as a useful resource is declining day and night. Coal gangue is industrial waste, and it is convenient to get nearby materials. The use of coal gangue to fill the foundation has a short construction period, low cost, and high economic benefits for areas where construction materials are not very abundant [32].

Coal gangue is a loose deposit with unique particle structure and composition. The physical and mechanical properties of coal gangue compaction are significantly different from other loose materials. The theoretical research on DC method is in development stage and is primarily carried out on soil with varying traits. There remains a lack of understanding regarding the theoretical research on gangue filling.

In order to obtain the construction technology for complex and deep coal gangue filling constructed by DC and obtain the bearing characteristics of coal gangue filling after DC, it is necessary to select a representative test site to carry out a field study on the improvement of a coal gangue filling using DC.

## 2. Test site and test equipment

### 2.1 Test site selection

**Fig 1:**
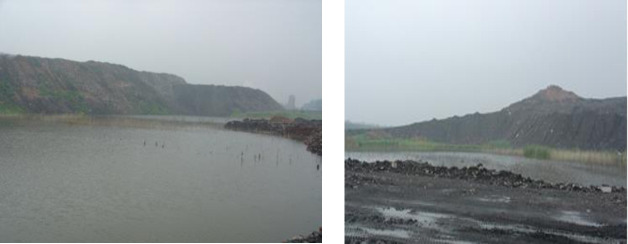
Test site before the DC test.

A site near the gangue hill pond was selected as the test site for improving the deep complex coal gangue filling using the DC method (Fig 1). The maximum backfill depth is in the range 6–8 m. The area of the test site is mainly determined according to the distance between ramming points, the number of ramming points, and four different tamping energy types. The pond is 50–80 m in length and 30–50 m in width, meeting the requirements of the test site. To ensure the test results are representative, the equipment used in this test site is the same as the equipment used in large-scale construction sites. In addition, the test site is close to the gangue hill; hence, it is convenient to construct a filling pond.

### 2.2 Test site treatment

**Fig 2:**
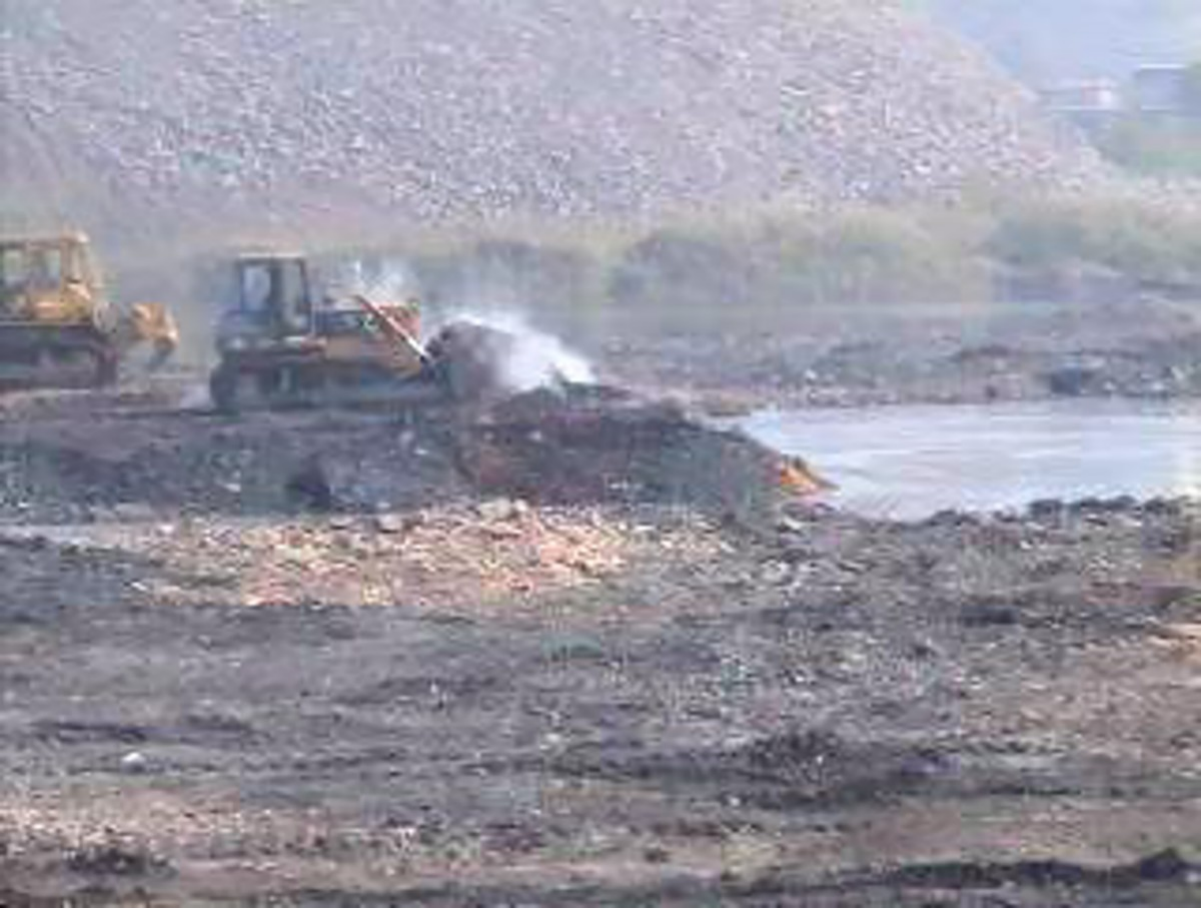
Bulldozer pushes coal gangue to fill pond.

Since the pond contains a large amount of water and clay thickness on the bottom is approximately 0.5 m, quickly draining water is difficult. The test site is filled using a high-filling method using the following procedure. First, the water in the pond was drained as much as possible. Then dumping coal gangue and squeezing silt method was used to deal with the pond base. The method of dumping coal gangue and squeezing silt is to fill the pond with coal gangue to squeeze out the silt. Large pieces of coal gangue were selected to fill the pond until the fill height was at the surface of the water. Ordinary coal gangue was used to fill the pond with a bulldozer until the height reached the design elevation + 1.0 m (1.0 m for the expected settlement). The coal gangue was rolling compacted during the filling process (Fig 2).

### 2.3 Field test equipment selection

The main construction equipment required for the field test includes dynamic crusher, rammer, heavy bulldozer (TY 230), and dump truck. A crawler type dynamic crusher with a lifting capacity of 50 t and a lifting height of 20m (equipped with automatic decoupling device) was used in this test. The main rammer has a weight of 25 t, a height of 1.6 m, a diameter of 2.3 m, and a bottom area of 4.15 m^2^. The full rammer has a weight of 10 t, with the same of diameter and bottom area as the main rammer. The bulldozer named TY230. The dump truck has a load of 20 t. The last pass was performed across the entire site.

## 3. Field test

### 3.1 Field test design for dynamic compaction

**Fig 3:**
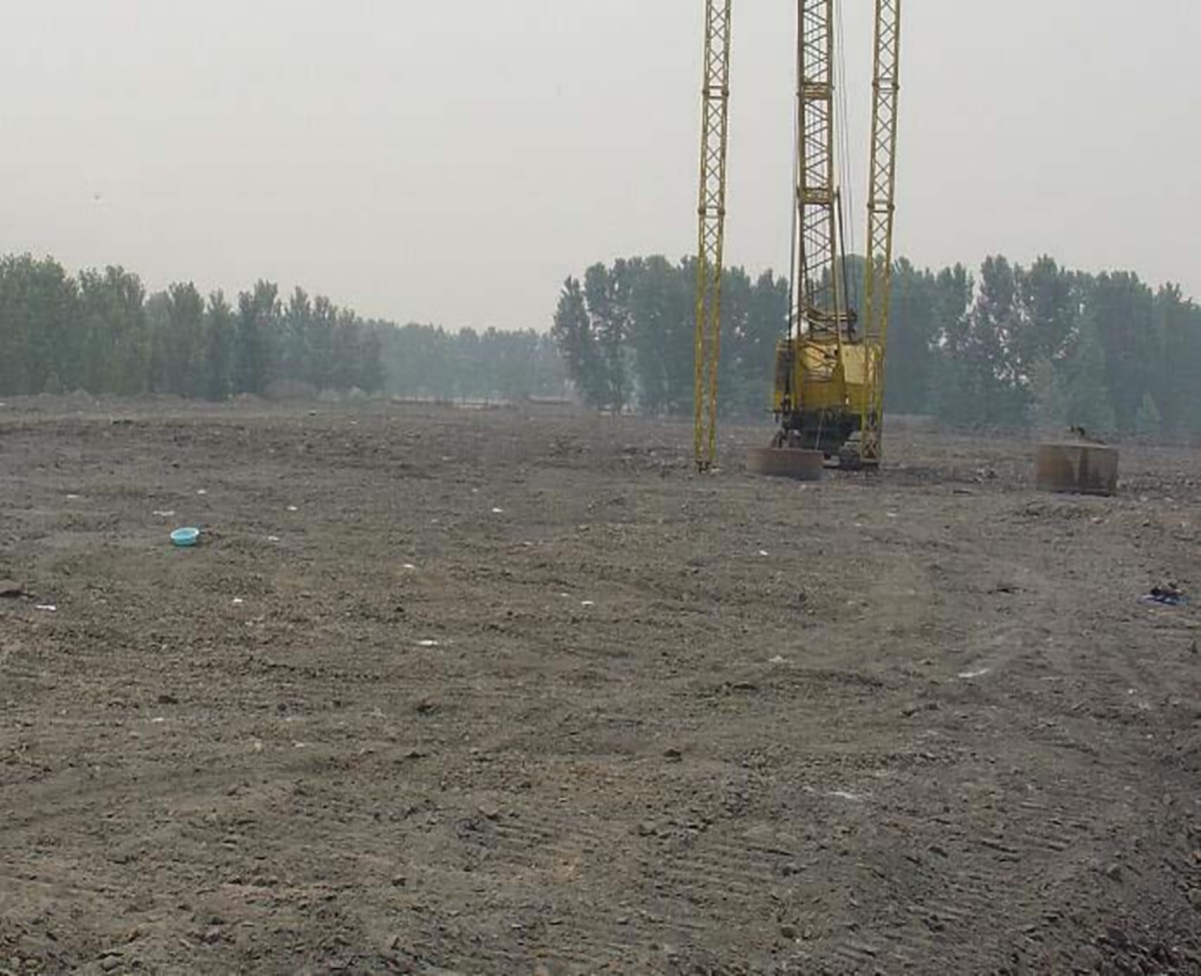
Test site.

**Fig 4:**
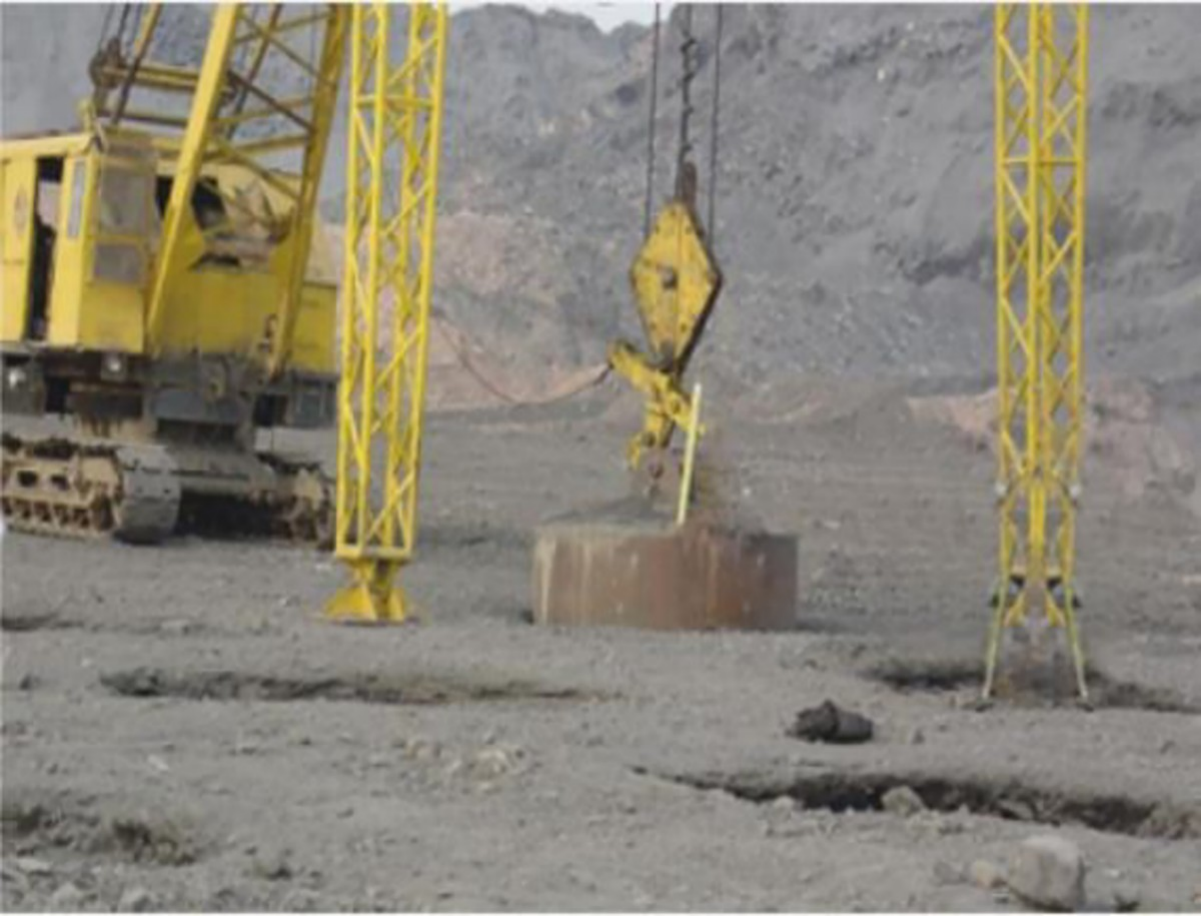
DC field test.

The main parameters of DC method include the hammer weight (m), falling distance (h), number of tamping blows(n), improving depth (H), and ramming point distance (L). According to laboratory model tests [33, 34], the effective improving depth and optimum number of tamping blows for coal gangue filling under different tamping energy were obtained. The field test was conducted based on the results of laboratory simulation tests to study the relationship between the tamping energy, effective improving depth, and optimum number of tamping blows under actual engineering conditions. Four different tamping energy values (2000, 2500, 3000, and 3500 kN.m) were used in this study. The test site and DC field test are shown in Figs 3 and 4, respectively.

### 3.2 Layout of ramming points

**Fig 5:**
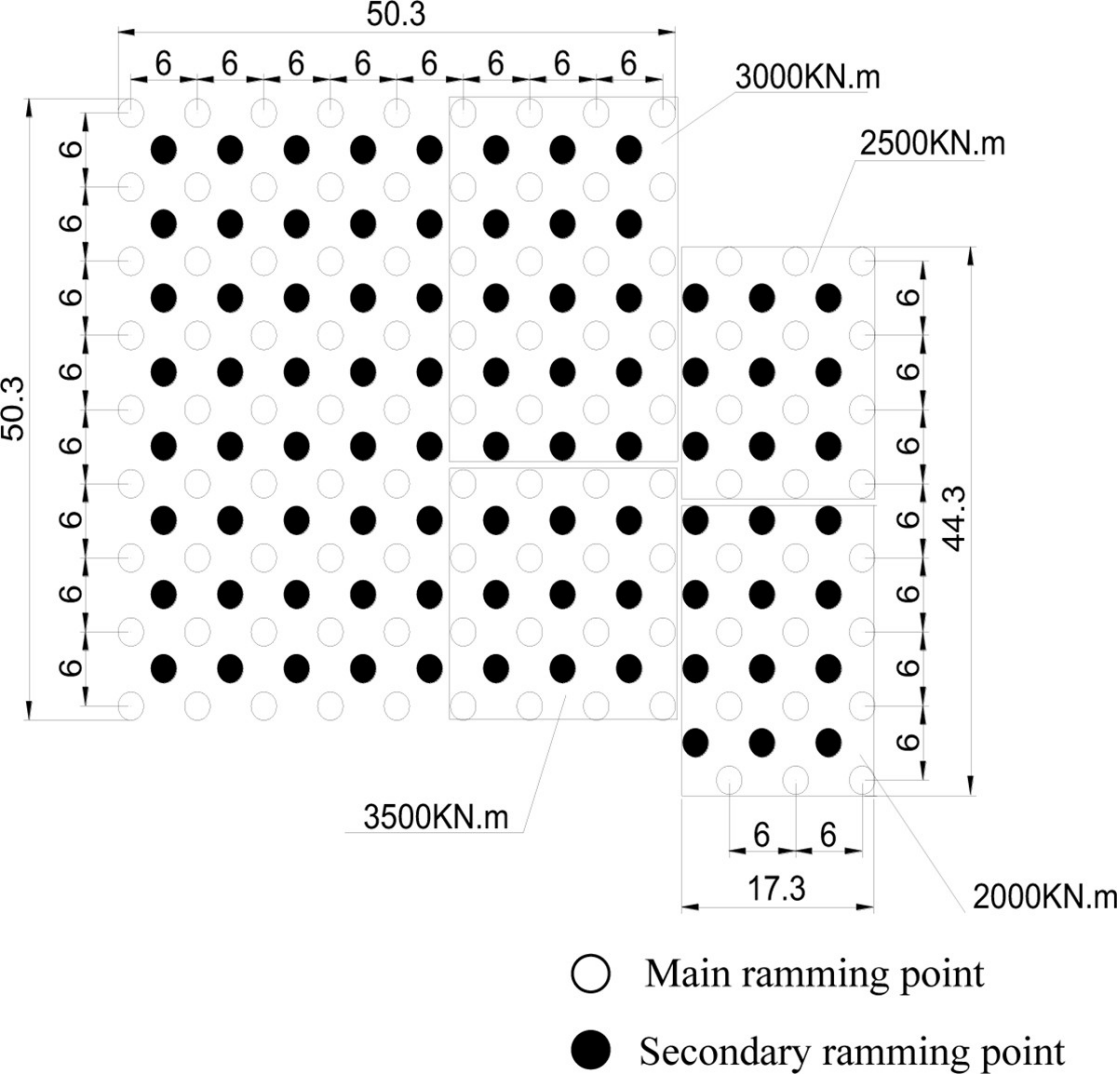
Layout of the ramming points in field test.

The test site area includes two parts, with the areas of 50.3 m× 50.3 m and 44.3 m×17.3 m. The main ramming point spacing is 6 m. The secondary ramming point spacing is 6 m too, as shown in Fig 5.

### 3.3 Method for measuring movement and deformation of coal gangue filling by DC

**Fig 6:**
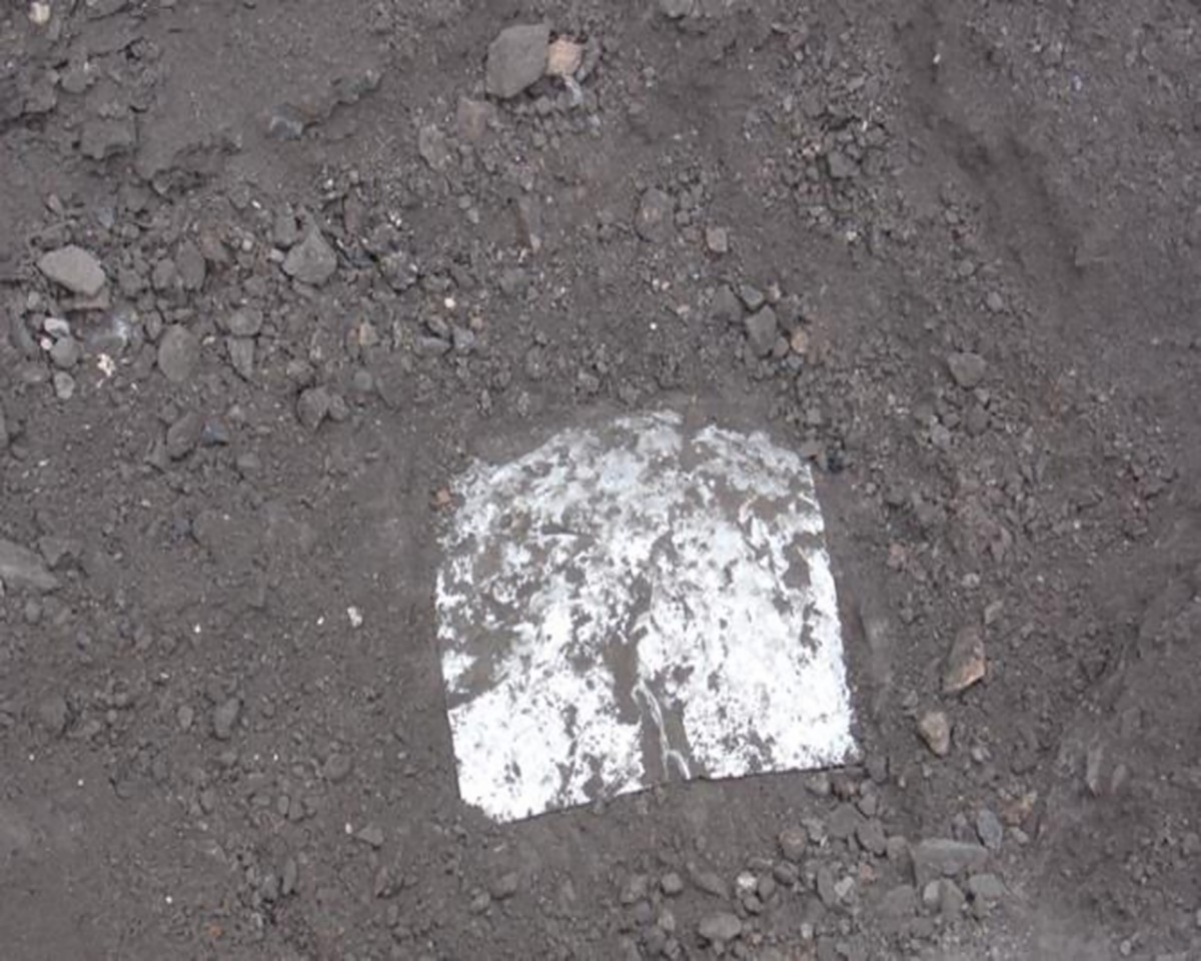
Location of steel plate.

**Fig 7:**
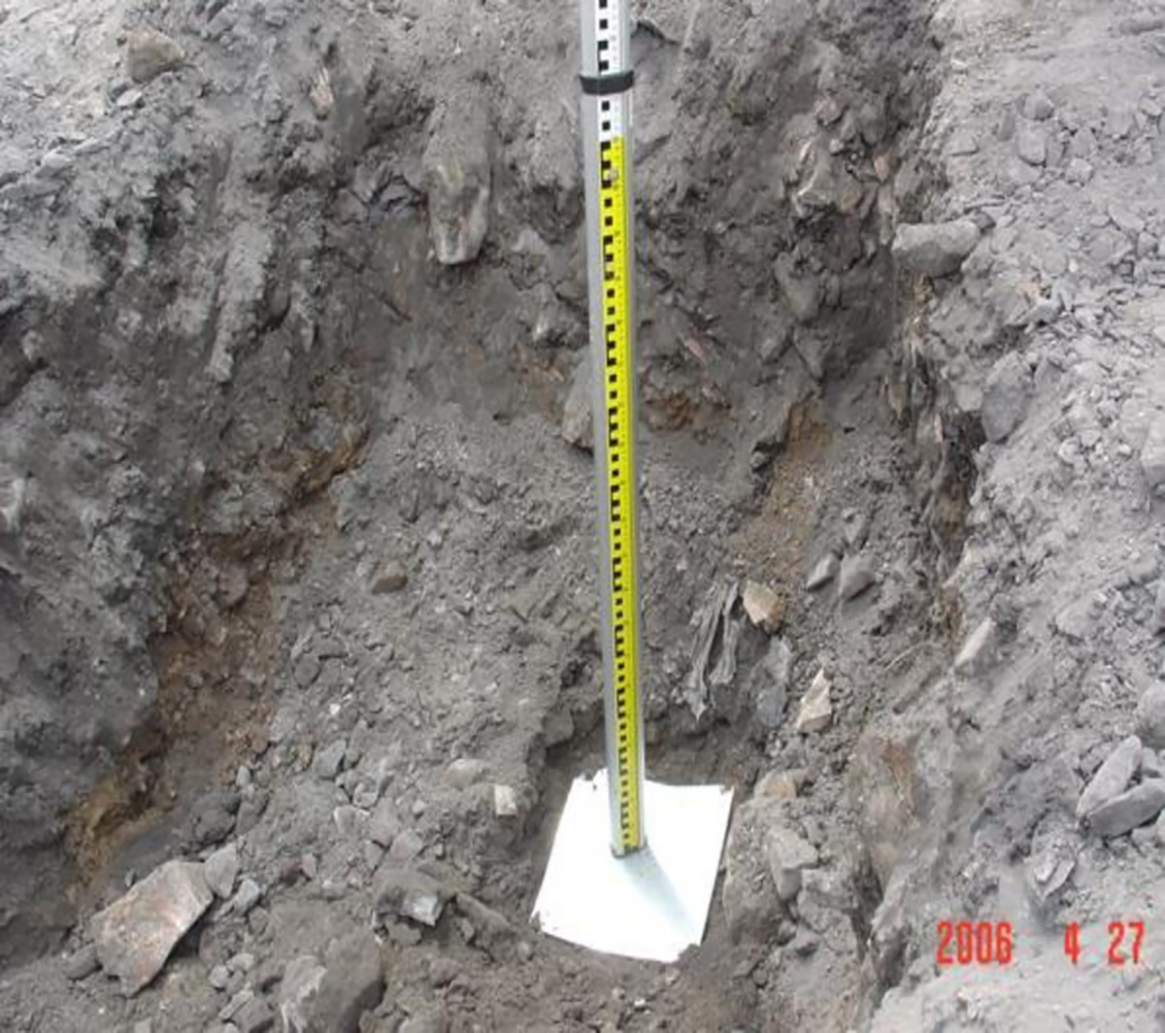
Measure the tamping settlement.

In order to examine the deformation of coal gangue filling under different tamping energy levels, excavation was carried out in the 2000, 2500, and 3000 kN.m tamping energy test areas, steel measuring points were embedded at different depths, and initial elevations were measured. After DC, the final elevations of the measuring points were measured. The difference between the final and initial elevations is the tamping induced deformation. Measuring points were embedded in the three different tamping energy test areas, separately (Figs 6 and 7).

### 3.4 Method of coal gangue particle size distribution after DC

The measuring points for measuring tamping induced grain breakage are the same as the deformation measuring described in section 3.3. After tamping, the coal gangue was excavated, and screening test at the construction site was conducted and coal gangue gradation curves were obtained for different tamping energies.

### 3.5 Tamping blows n and tamping passes N

The number of tamping blows n was determined according to the relationship between the tamping blows and tamping induced deformation obtained in the field test. To determine the number of tamping blows, the average tamping settlement induced by the last two tamping blows should not be greater than 50mm, no large uplift should occur around the tamping point, and deep tamper holes should be avoided (because the hammer is difficult to lift in a deep tamper hole).

The whole DC test includes three passes. During the dynamic compaction construction, the first pass is performed in accordance with the designed spacing, the second pass is performed in the middle of the designed spacing, and the third pass is performed across the entire site. Each pair of tamping points has an overlap of 1/4 the tamper size. The main ramming and the secondary ramming have the same tamping energy, which is the test tamping energy. The full ramming tamping energy is 1000 kN.m.

### 3.6 Field test results and analysis

#### 3.6.1 Tamping induced settlement and the optimum tamping blows

**Fig 8:**
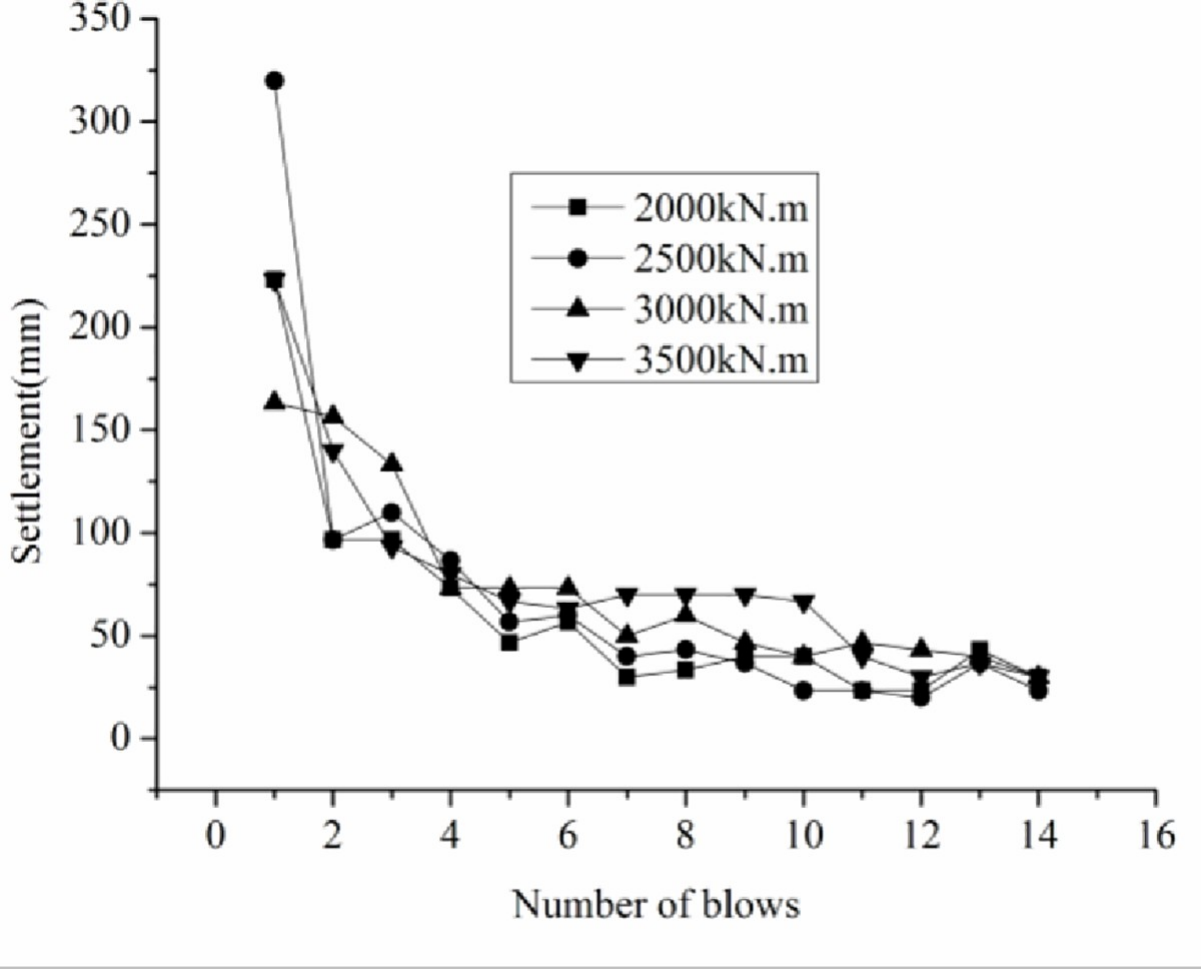
The tamping settlement curve with varying tamping energy.

**Fig 9:**
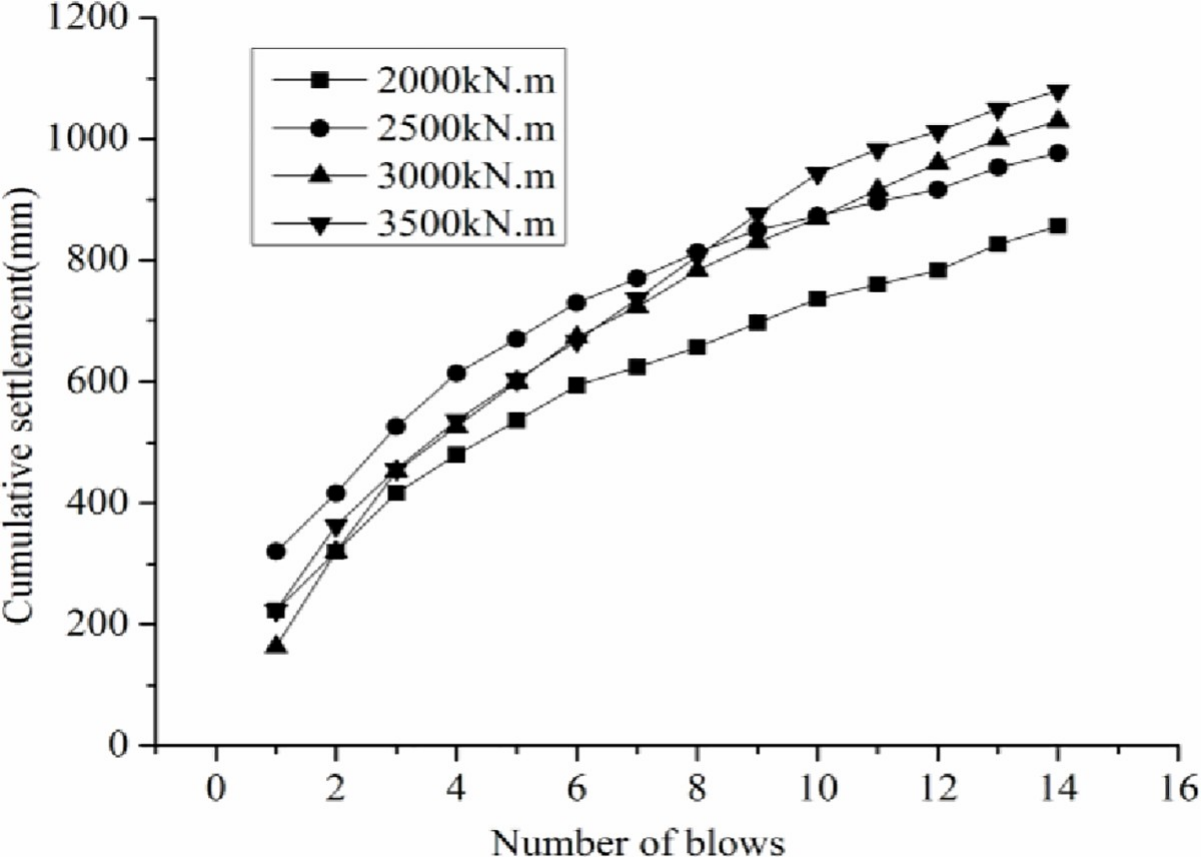
The cumulative tamping settlement curve with varying tamping energy.

Three measuring points were selected at the top of the hammer, and their initial elevations were measured before tamping. After each blow, the elevations of the three points were measured again, and the average elevation was calculated. The difference in the average elevation between individual tamps is the tamping induced settlement. Fig 8 shows the tamping settlement curve with varying tamping energy, and the cumulative tamping induced settlement curve with varying tamping energy is shown in Fig 9.

When DC method was used for continuous tamping, the first tamping blow inducts the largest settlement, approximately 160–320 mm (Fig 8). With increasing tamping blow, the single tamping induced settlement decreased. Taking a tamping energy of 2000 kN.m as an example, for the first tamping blow, the settlement is 220 mm, whereas the settlements induced by the second, third, fourth, and fifth tamping blows are 100, 100, 70, and 50 mm, respectively, which is likely attributed to the fact that the coal gangue filling is loose and un-homogeneous at the beginning of tamping, and a large settlement was induced by tamping. With increasing tamping blow, large particles were broken and macropore was filled with small and broken particles, resulting in a more compacted filling and reducing the single tamping settlement.

The cumulative tamping induced settlement increases with increasing tamping blows (Fig 9). The cumulative tamping induced settlement linearly increases with increasing tamping energy. Under a tamping energy of 3000 kN.m, the cumulative settlement is greater than 1.0m. The optimum tamping blow increases with increasing tamping energy. The optimum tamping blows for tamping energy of 2000, 2500, 3000, and 3500 kN.m are 7, 7, 9, and 11, respectively.

For the small-scale model test, when the number of tamping blows is 7, the average settlement of the last two blows is less than 50 mm, indicating that the optimal tamping blows are 7. For the large-scale model test, when the tamping blows are 9, the average settlement of the last two blows is less than 50 mm, indicating that the optimal tamping blows are 9. Therefore, the best number of tamping blows is 7–9 [33]. The field test results and laboratory simulation test results are basically the same.

#### 3.6.2 Tamping induced deformation

**Fig 10:**
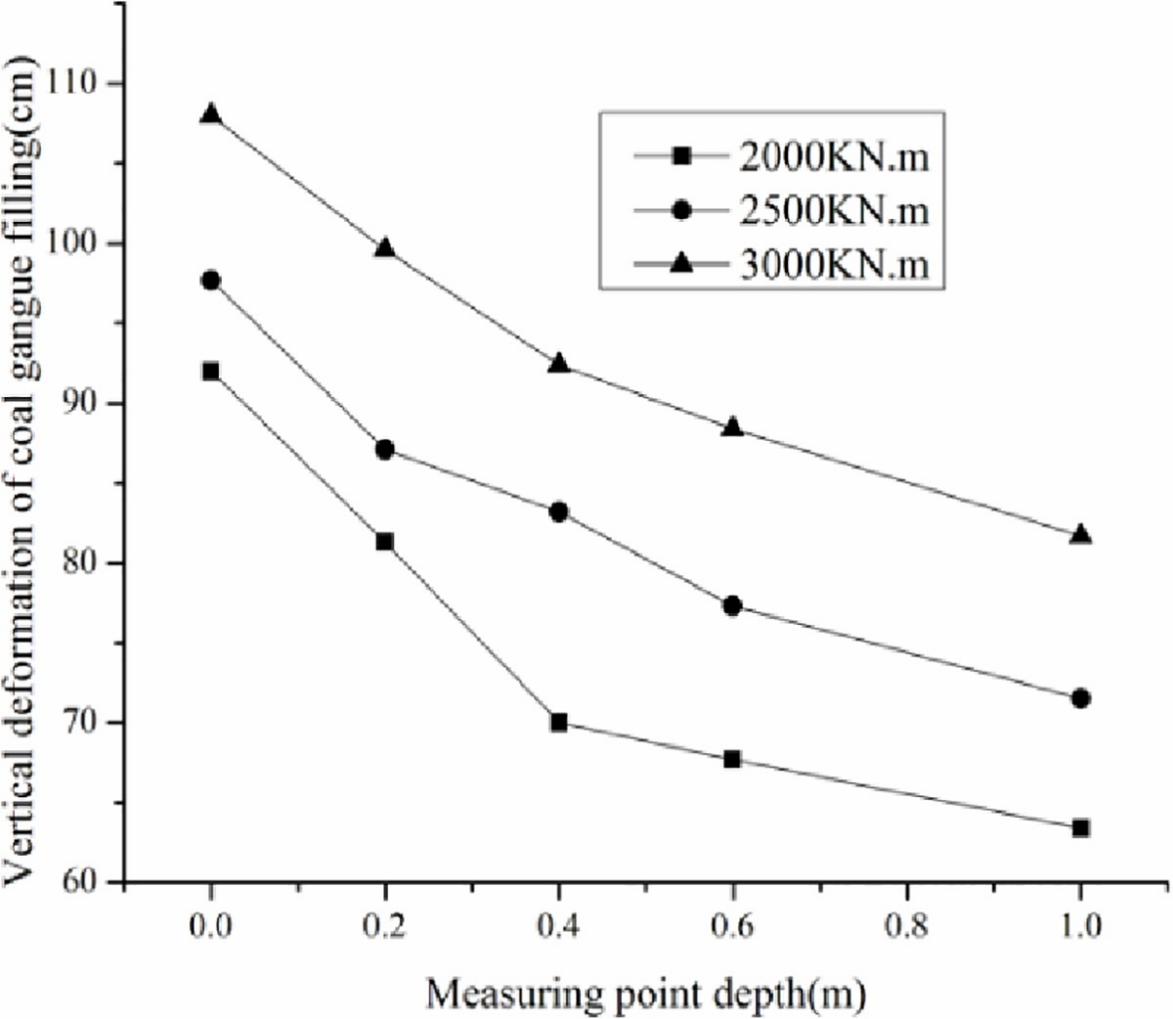
Vertical deformation curve of the goal gangue filling under different tamping energy.

The effective improving depth H of the gangue filling is affected by the tamping energy. The effective improving depth in the field can be determined by the range of the deformation induced by tamping. The area of the coal gangue filling where the coal gangue is crushed and the density is increased after tamping is defined as the effective improving depth. The vertical deformation of the coal gangue filling at different depths with different tamping energy is shown in Fig 10.

Because of DC, the coal gangue filling is crushed and compacted, and the ground surface subsidence and internal movement of the filling are observed. The moving deformation follows an exponential distribution with depth. The effective improving depth under field conditions was determined by the range of movement and deformation of coal gangue filling. The area of relative movement and deformation of the gangue filling before and after DC is the area, where the density of coal gangue is increased by DC, and the depth of this area is the gangue filling DC effectively strengthens the depth. According to this principle, when tamping energy is equal to 2000 kN.m, the effective improving depth H is 5.38 m, when tamping energy is 2500 kN.m, H is 7.02 m, and when tamping energy is 3000 kN.m, H is 7.92 m. According to filling depth conditions used in this study, a tamping energy greater than 3000 kN.m should be used in the actual construction process.

#### 3.6.3 Particle gradation analysis of coal gangue filling

**Fig 11:**
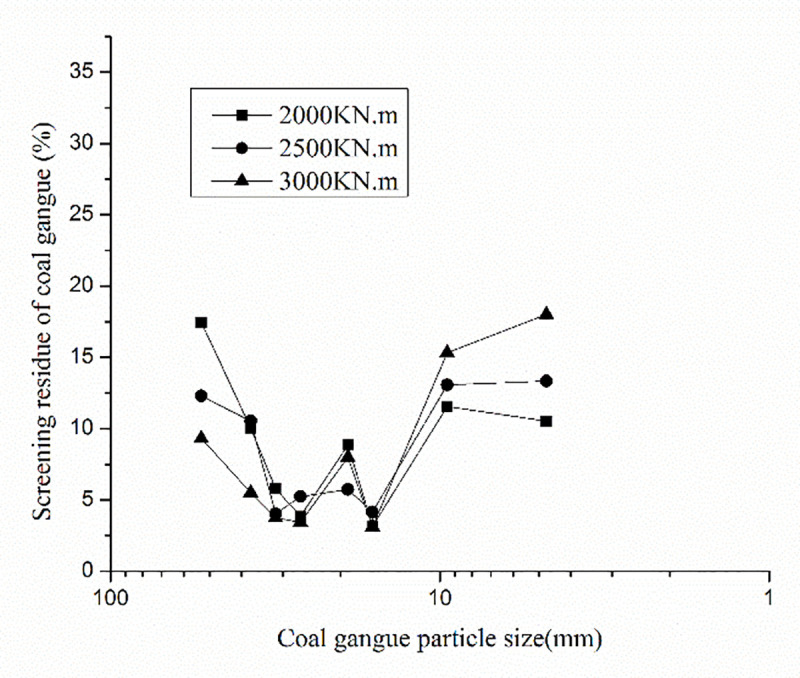
Comparison of residual curve of coal gangue particle sieve points by different tamping energy.

**Fig 12:**
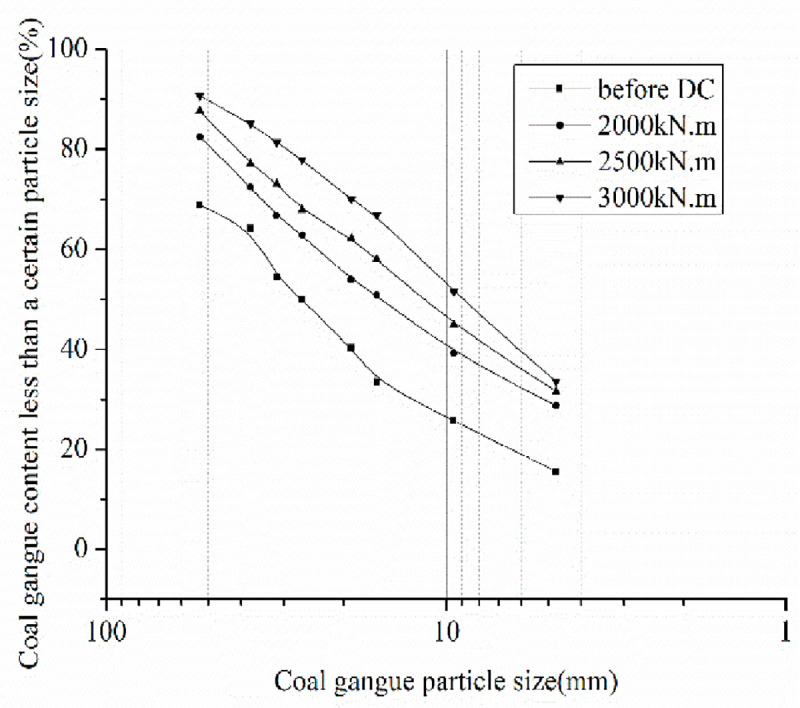
Passage rate curve of coal gangue particles after different tamping energy.

After DC, the excavated coal gangue was evenly mixed, and a representative portion was subjected to an on-site screening test. The coal gangue contains fine particles with a particle size less than 4.75mm, coarse particles larger than 4.75mm. k_w_ is the coal gangue fine-rough grain ratio. The coal gangue fine-rough ratios after DC are 0.4032, 0.4616, and 0.5038 for tamping energies of 2000, 2500, and 3000kN.m, respectively. The grain gradation curves under different tamping energies are shown in Figs 11 and 12.

The pass rate of coal gangue gradation increased, the coarse grain composition significantly reduced, and the fine particle composition increased, resulting in a good gradation (Figs 11 and 12). After DC, coal gangue fine-rough ratio increases greatly and shows an increasing tendency with increasing the tamping energy. Under a tamping energy of 3000 kN.m, the coal gangue fine-rough ratio is larger than 0.5, resulting in good mechanical behavior of the coal gangue filling. In order to increase the crushing rate of coal gangue coarse particles during tamping, improve the fine-rough ratio, and improve the physical and mechanical properties of coal gangue filling, the single tamping energy should be greater than 3000kN.m.

According to the change in the gradation curve after the indoor model test, it can be seen that the large-size coal gangue crushing caused by DC is obvious, the proportion of fine particles increases greatly, the gradation situation significantly improved, and the improvement effect of DC is obvious. Before DC, the average values of unevenness coefficient C_u_ and curvature coefficient C_c_ were 5.47 and 0.83, respectively, indicating that the coal gangue gradation before DC was poor. After DC, for the tamping energy of 2000 kN.m, the range values of the unevenness coefficient C_u_, and the curvature coefficient C_c_ are in the ranges 6.22–7.2 and 1.11–1.24, respectively, with the average values 6.89 and 1.12 for the tamping energy of 3000 kN.m. The range values of the unevenness coefficient C_u_ and the coefficient C_c_ are in the ranges 5.23–5.50 and 1.09–1.14, with the average values of 5.40 and 1.10, respectively. It shows that the gradation of coal gangue after DC significantly improved, meeting the requirements of good gradation [35].

### 3.7 Field test of bearing capacity of coal gangue construction site

When DC test is completed, the static load test and heavy dynamic penetration test (N_63.5_) were carried out to assess the effect of DC on the mechanical behavior of the coal gangue filling following the Chinese Standard "Technical code for ground treatment of building" JGJ79-2002 and "Code for design of building foundation" GB50007-2002. The coal gangue filling bearing capacity after DC was measured using the slow maintenance load method. The compactness and uniformity of the coal gangue filling after DC were analyzed using heavy dynamic penetration test (N_63.5_) following the standard "Geotechnical Engineering Survey" GB50021-2001.

The dynamic penetration test (N_63.5_) is composed of a drop hammer, a probe, and a probe rod. Among them, the drop weight is 63.5 kg, the drop distance is 0.76 m, the probe diameter is 74 mm, the cross-sectional area is 43 cm^2^, the cone angle is 60°, the probe diameter is 42.5 mm, and N_63.5_ is the number of strokes with a penetration depth of 10 cm. The hammer penetration is carried out continuously, with the hammering speed of 15–20 hits/min. The test will be stopped when the hammer has more than 50 hits for 3 consecutive times.

#### 3.7.1 Test equipment

**Fig 13:**
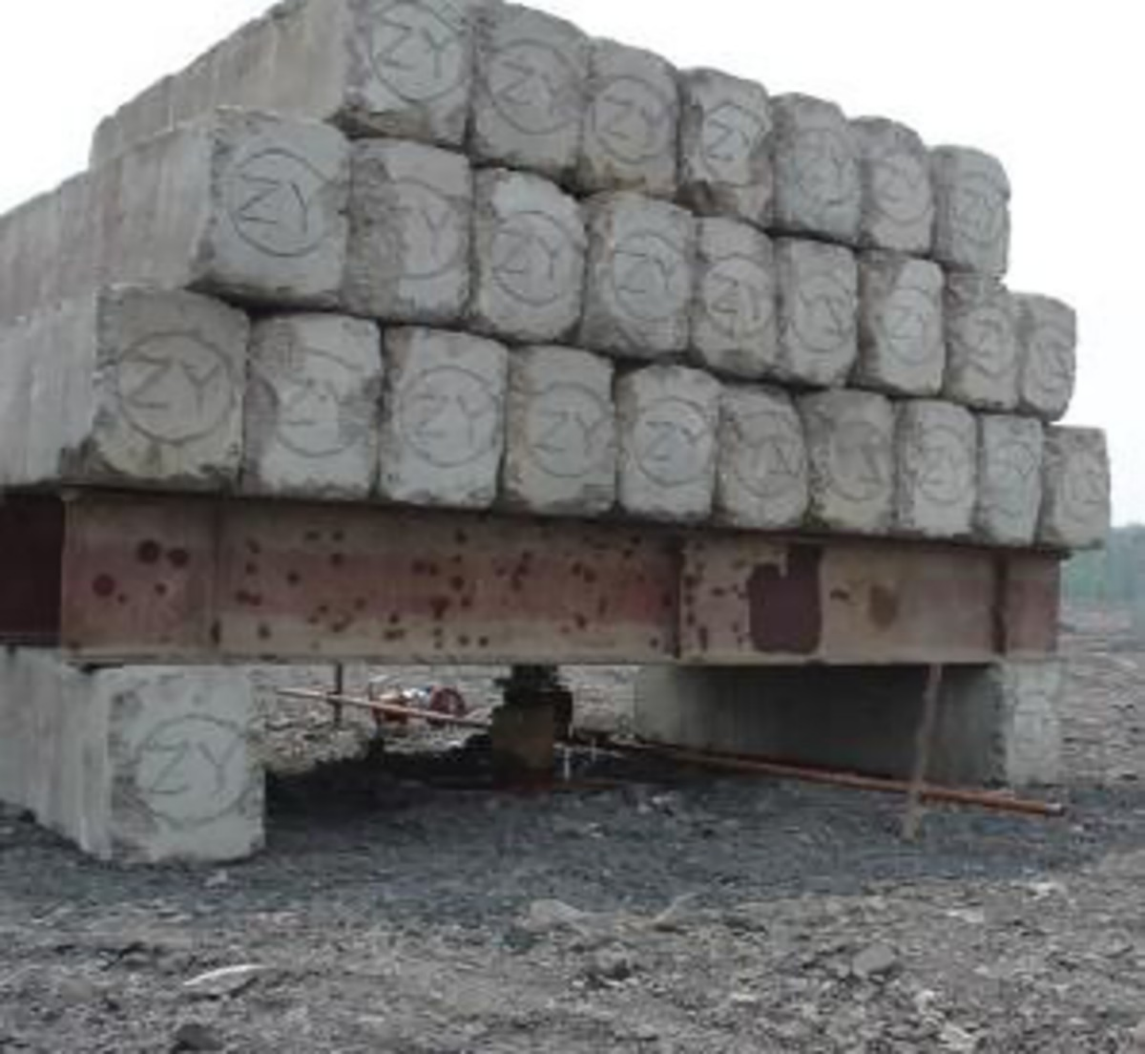
The static loading test.

The static loading test equipment includes a static loading automatic test system(JCQ-503A), a pressure sensor(ZZY-474), two displacement sensors (MS-50), a 200 ton-capacity jack, a steel beam static loading reaction device (weight prefabricated cement block of 84 t), and a square bearing plate with dimensions of 1.0 m×1.0 m. The loading level is 10, and the load of each level is 70kPa (Fig 13).

#### 3.7.2 Field test layout of test points

Eight measuring points were set for the static loading test with two measuring points for each area tamped under a certain tamping energy (Table 1).

**Table 1:** Measuring points for the static loading tests with different tamping energy.

Tamping energy kN.m	2000	2500	3000	3500
number	2	2	2	2
Measuring point number	J2-1, J2-2	J2.5–1, J2.5–2	J3-1, J3-2	J3.5–1, J3.5–2

Sixteen measuring points were set for heavy dynamic penetration test with three measuring points for each area tamped using certain tamping energy. Four measuring points were used for the un-compacted area. Details are listed in Table 2:

**Table 2:** Measuring point layout for heavy dynamic penetration test.

Measuring point partition	Tamping energy kN.m	number	Measuring point number
Improving area	2000	3	D2-1/5.80m, D2-2/5.80m, D2-3/ 6.10m
	2500	3	D2.5-1/6.10m, D2.5-2/6.00m, D2.5-3/6.20m
	3000	3	D3-1/6.00m, D3-2/6.00m, D3-3/5.80m
	3500	3	D3.5-1/5.80m, D3.5-2/5.40m, D3.5-3/ 5.60m
Unreinforced area	Pond section	2	D0-1/6.10m, D0-2/6.40m
	Gangue hill	2	D0-3/5.60 m,D0-4/5.90 m

**Fig 14:**
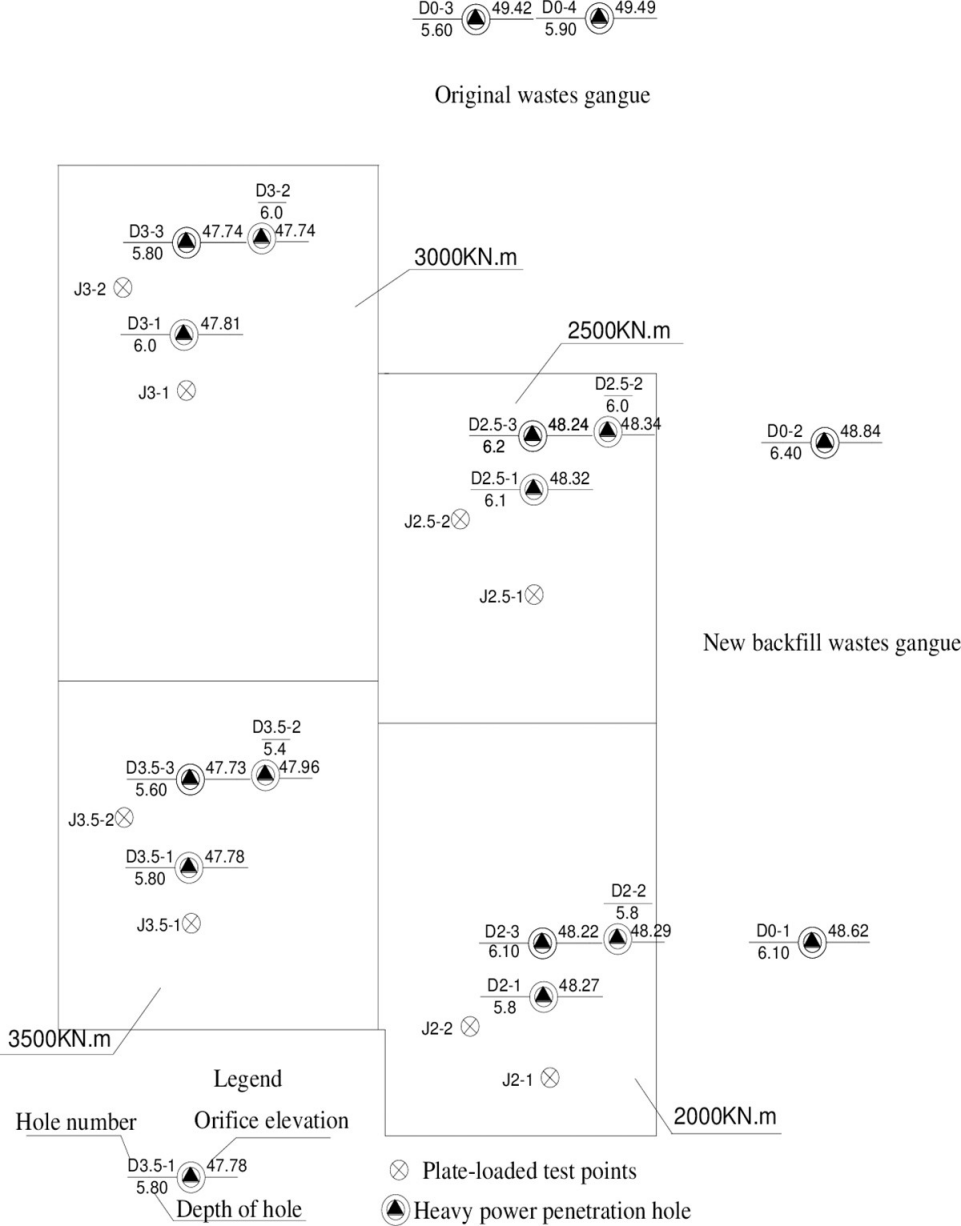
Layout of test points for the static load loading test and heavy dynamic penetration test.

Fig 14 shows the site measuring points.

#### 3.7.3 Field test results and analysis

**Fig 15:**
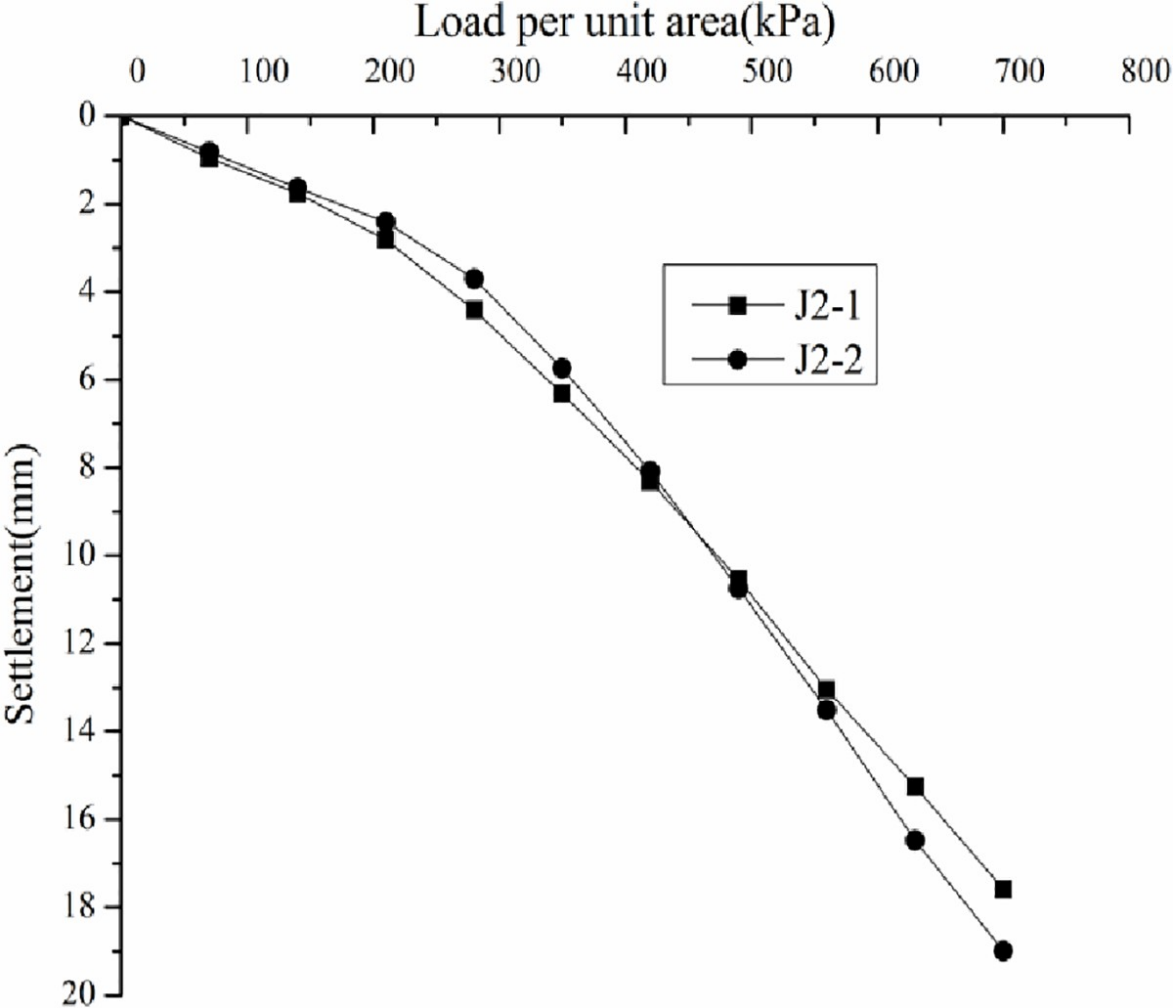
Load-–settlement curves for the plate loading tests of J2.

**Fig 16:**
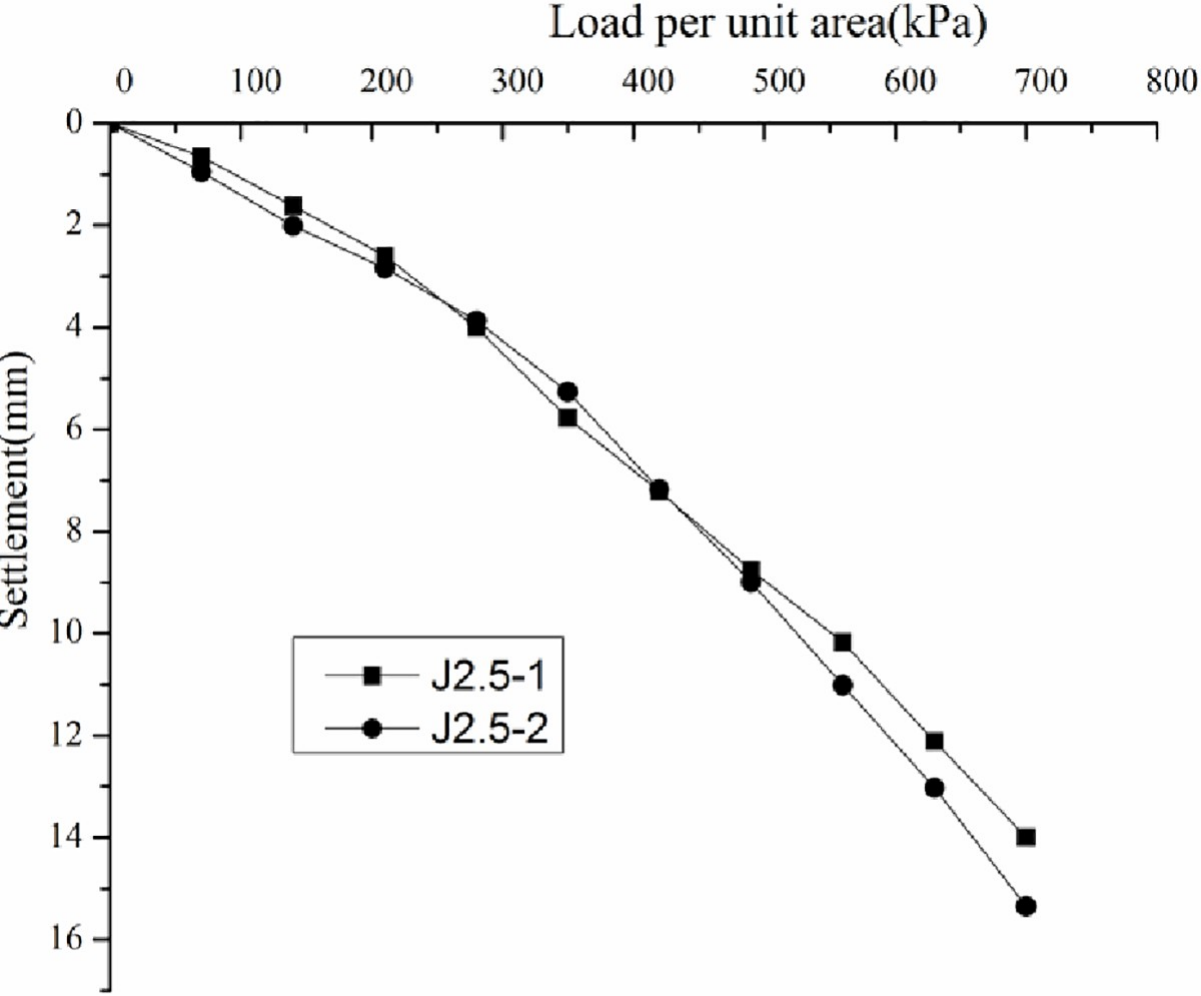
Load–settlement curves for the plate loading tests of J2.5.

**Fig 17:**
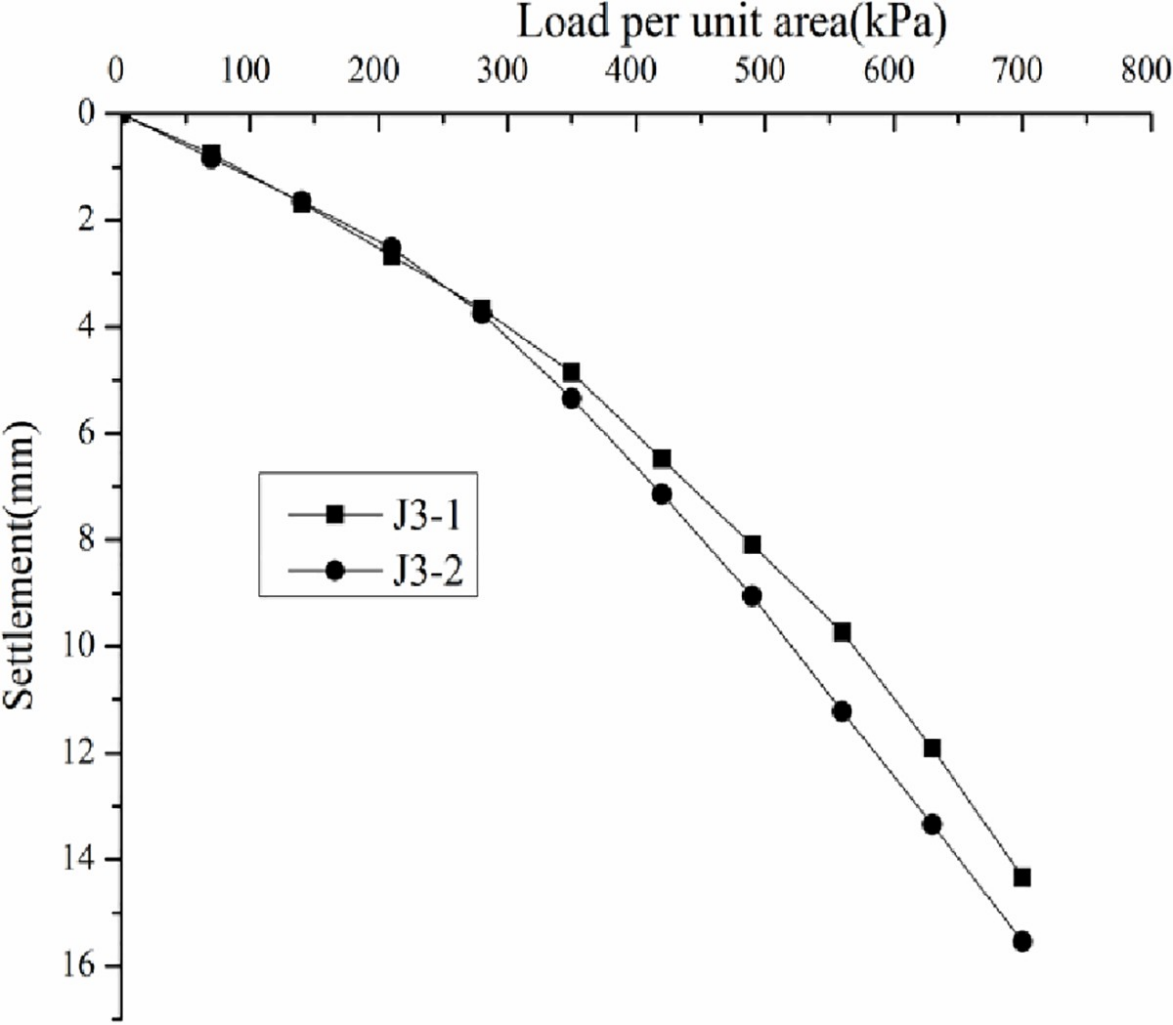
Load–settlement curves for the plate loading tests of J3.

**Fig 18:**
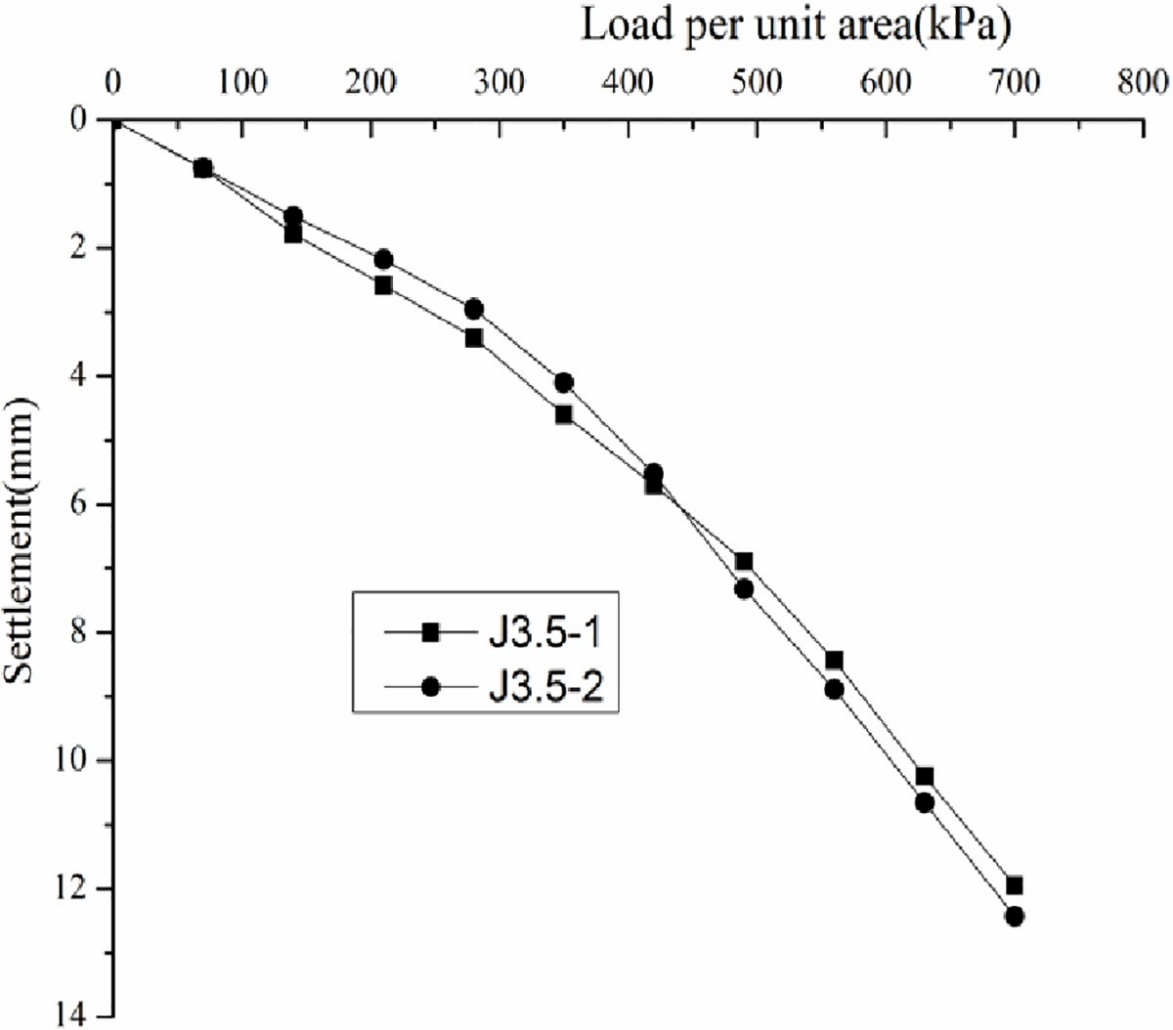
Load–settlement curves for the plate loading tests of J3.5.

*3*.*7*.*3*.*1 Analysis of static loading test results*. Load–settlement curve (p–s curve) was drawn according to the static loading test results (Figs 15–18).

Figs 15–18 do not show any obvious yield inflection point in the p–s curves under the maximum load of 700 kPa. According to the Standard "Technical Code for ground treatment of building" JGJ79-2002, the characteristic values of bearing capacity of each measuring point should be determined following the relative deformation value, which is s/b = 0.01, where b = 1000 mm is the width of the pressure plate. When the settlement was equal to 10mm, the ultimate bearing capacity can be obtained (Table 3).

**Table 3:** Bearing capacity and characteristic values of the coal gangue site treated by the DC method.

Tamping energy kN.m	Measuring point	Final load/kPa	Maximum settlement/mm		Ultimate bearing capacity/kPa		Bearing capacity characteristic value/kPa
2000	J2-1	700	17.58	18.29	473	472	350
	J2-1	700	18.99		470		
2500	J2.5–1	700	13.99	14.67	552	539	350
	J2.5–2	700	15.35		525		
3000	J3-1	700	14.33	14.94	569	545	350
	J3-2	700	15.54		521		
3500	J3.5–1	700	11.95	12.19	621	614	350
	J3.5–2	700	12.43		607		

According to the Standard "Technical Code for ground treatment of building" JGJ79-2002, the bearing capacity characteristic value determined by the relative deformation value should not be greater than half the maximum loading pressure. The ultimate bearing capacity in Table 3 is greater than half the maximum loading pressure 350 kPa, hence the three bearing capacity characteristic values were determined to be 350kPa.

By analyzing Figs 15–18 and Table 3, the following conclusions can be drawn. The p–s curve of coal gangue filling has no yield inflection point. The maximum settlement decreases with increasing tamping energy. Increasing the tamping energy can increase the ultimate bearing capacity of the gangue filling and reduce filling settlement. According to the static loading test, the maximum loading is 700 kPa, and the bearing capacity of coal gangue filling is controlled by deformation. For four different tamping energy values, the characteristic value of bearing capacity is at least 350 kPa.

The p-s curve for untreated ground (before DC) is not presented. However, the bearing capacity can be obtained by simulated load test method, which is about 100kPa. The bearing capacity of coal gangue filling is about 3.5 times higher than that before DC. After DC, the coal gangue filling has good stability, strong resistance to deformation, and high bearing capacity. According to the requirements for bearing capacity and deformation of multi-story buildings in the demonstration project, when the tamping energy is greater than 3000 kN.m, the bearing capacity and deformation can meet the building requirements of the multi-story buildings.

*3*.*7*.*3*.*2 Analysis of heavy dynamic penetration test results*. The standard penetration number of coal gangue is small before DC, indicating the coal gangue filling is loose (Table 4).

**Table 4:** Heavy dynamic penetration test results.

Project	Before DC		Tamping energy kN.m			
	Original site	New site	2000	2500	3000	3500
Improvement depth /m			3.7–4.0	3.5–4.1	3.7–3.8	>5.4–5.8
Hit range / hit	5–20	0.5–7	>20	>20	>20	>20
Average hit number	11.05	3.05	38.2	39.4	42.4	47.6

According to “Code for design of building foundation”, GB50007-2002, the bearing capacity of crushed stone foundation is shown in Table 5. The density of crushed stone foundation is listed in Table 6.

**Table 5:** Relationship between N_63.5_ and bearing capacity.

N_63.5_	3	4	5	6	8	10	12	14	16
σ_0_(kPa)	140	170	200	240	320	400	480	540	600
N_63.5_	18	20	22	24	26	28	30	35	40
σ_0_(kPa)	660	720	780	830	870	900	930	970	1000

**Table 6:** The density of crushed stone foundation.

N_63.5_	density
N_63.5_≤5	loose
5<N_63.5_≤10	Slightly density
10<N_63.5_≤20	Middle density
N_63.5_>20	density

The average standard penetration number of the original site is larger than that of the new coal gangue site, indicating the coal gangue that has been stripped from the site with a certain preload effect on the lower coal gangue filling. However, the density of the original site is still relatively low and must be treated by DC. The standard penetration number increased after DC. When the tamping energy is 2000 kN.m, the standard penetration number is 38.2, and the standard penetration numbers are 39.4, 42.4, and 47.6 for tamping energy of 2500, 3000, and 3500 kN.m, respectively. The standard penetration number increased with increasing tamping energy. After DC, the density also increased with increasing tamping energy.

The heavy dynamic penetration test results show that before DC, the coal gangue particle structure is loose, and after DC, it becomes dense, and the corresponding bearing capacity improves. The demonstration project should be treated using a tamping energy greater than 3000 kN.m to ensure an adequate filling improving depth.

## 4. Conclusions

The primary conclusions of the paper are as follows:

1. The DC method is a reliable method for improving complex and deep coal gangue filling. After DC, the physical and mechanical properties of the gangue filling improved, bearing capacity increased, and the resistance to deformation enhanced. The mechanical behavior can meet the necessary requirements after DC.
2. Tamping energy can induce a shock wave, breaking coal gangue particles and improving coal gangue gradation. With increasing tamping energy, the shock wave was increased, increasing the effective improving depth. When tamping energy is greater than 3000 kN.m, the effective improving depth is approximately 6–8 m and the fine-rough ratio is greater than 0.5, improving the mechanical behavior of the coal gangue filling.
3. With increasing number of tamping blows, the single tamping blow induced settlement decreases and begins to stabilize. The optimum number of tamping blows increases with increasing tamping energy and the optimum number of tamping blows is 9–11 at the tamping energy greater than 3000 kN.m.
4. Coal gangue filling bearing capacity and anti-deformation ability increase with increasing tamping energy. When the tamping energy is greater than 3000 kN.m, the bearing capacity can reach at least 350 kPa.
